# Association between skeletal muscle mass and serum concentrations of lipoprotein lipase, GPIHBP1, and hepatic triglyceride lipase in young Japanese men

**DOI:** 10.1186/s12944-019-1014-7

**Published:** 2019-04-04

**Authors:** Ryutaro Matsumoto, Katsuhiko Tsunekawa, Yoshifumi Shoho, Yoshimaro Yanagawa, Nobuo Kotajima, Shingo Matsumoto, Osamu Araki, Takao Kimura, Katsuyuki Nakajima, Masami Murakami

**Affiliations:** 1 Department of Clinical Laboratory Medicine, Gunma University Graduate School of Medicine, Maebashi, 371-8511 Japan; 2 Faculty of Education, Ikuei University, Takasaki, 370-0011 Japan; 3 School of Medical Technology, Faculty of Health Science, Gunma Paz University, Takasaki, 370-0006 Japan; 4 Graduate School of Health and Sport Science, Nippon Sport Science University, Yokohama, 227-0033 Japan

**Keywords:** Skeletal muscle mass, Lipoprotein lipase (LPL), Glycosylphosphatidylinositol anchored high-density lipoprotein binding protein 1 (GPIHBP1), Hepatic triglyceride lipase (HTGL), Thyroid hormone

## Abstract

**Background:**

Two important regulators for circulating lipid metabolisms are lipoprotein lipase (LPL) and hepatic triglyceride lipase (HTGL). In relation to this, glycosylphosphatidylinositol anchored high-density lipoprotein binding protein 1 (GPIHBP1) has been shown to have a vital role in LPL lipolytic processing. However, the relationships between skeletal muscle mass and lipid metabolism, including LPL, GPIHBP1, and HTGL, remain to be elucidated. Demonstration of these relationships may lead to clarification of the metabolic dysfunctions caused by sarcopenia. In this study, these relationships were investigated in young Japanese men who had no age-related factors; participants included wrestling athletes with abundant skeletal muscle.

**Methods:**

A total of 111 young Japanese men who were not taking medications were enrolled; 70 wrestling athletes and 41 control students were included. The participants’ body compositions, serum concentrations of lipoprotein, LPL, GPIHBP1 and HTGL and thyroid function test results were determined under conditions of no extreme dietary restrictions and exercises.

**Results:**

Compared with the control participants, wrestling athletes had significantly higher skeletal muscle index (SMI) (*p* < 0.001), higher serum concentrations of LPL (*p* < 0.001) and GPIHBP1 (*p* < 0.001), and lower fat mass index (*p* = 0.024). Kruskal–Wallis tests with Bonferroni multiple comparison tests showed that serum LPL and GPIHBP1 concentrations were significantly higher in the participants with higher SMI. Spearman’s correlation analyses showed that SMI was positively correlated with LPL (ρ = 0.341, *p* < 0.001) and GPIHBP1 (ρ = 0.309, *p* = 0.001) concentration. The serum concentrations of LPL and GPIHBP1 were also inversely correlated with serum concentrations of triglyceride (LPL, ρ = − 0.198, *p* = 0.037; GPIHBP1, ρ = − 0.249, *p* = 0.008). Serum HTGL concentration was positively correlated with serum concentrations of total cholesterol (ρ = 0.308, *p* = 0.001), low-density lipoprotein-cholesterol (ρ = 0.336, *p* < 0.001), and free 3,5,3′-triiodothyronine (ρ = 0.260, *p* = 0.006), but not with SMI.

**Conclusions:**

The results suggest that increased skeletal muscle mass leads to improvements in energy metabolism by promoting triglyceride-rich lipoprotein hydrolysis through the increase in circulating LPL and GPIHBP1.

## Background

The skeletal muscle is a major organ involved in systemic energy metabolism. As the population is aging rapidly, the increase in the number of patients with decreased skeletal muscle mass, called sarcopenia, is becoming an evident clinical problem. It is known that sarcopenia is associated with cardiovascular diseases and metabolic disorders, including dyslipidemia [1–5]; however, the detailed mechanism of metabolic dysfunction by sarcopenia remains to be clarified. It is believed that one way to prevent metabolic diseases is to maintain an appropriate amount of skeletal muscle through physical activities, such as exercise. However, there have been few reports on the effects of increasing skeletal muscle mass on lipid metabolism.

Two key enzymes involved in circulating lipid metabolism are lipoprotein lipase (LPL) and hepatic triglyceride lipase (HTGL) [6–9]. LPL has a major role in triglyceride (TG)-rich lipoprotein metabolism by catalyzing the hydrolysis of TG in chylomicrons and very low-density lipoproteins to form chylomicron and very low-density lipoprotein remnants, respectively [6, 7]. LPL is expressed and synthesized mainly in adipose and skeletal muscle tissue that store and consume fatty acids, respectively, and is translocated to the capillary lumen by glycosylphosphatidylinositol anchored high-density lipoprotein (HDL) binding protein 1 (GPIHBP1) [8]. GPIHBP1 anchors the LPL on the surface of capillary endothelial cells and has a crucial role in the lipolytic processing of TG-rich lipoproteins by LPL [7, 8]. Recent reports have demonstrated that GPIHBP1 autoantibodies and gene mutations cause hypertriglyceridemia [10–16]. Regarding HTGL, it has been known that it has a role in catalyzing the hydrolysis of the smaller remnants into low-density lipoprotein (LDL) and has a primary role in HDL metabolism [9, 17]. HTGL is synthesized in the liver and bound to heparin sulfate proteoglycans on surfaces of the liver sinusoidal capillaries [9, 17].

Thyroid hormone has a crucial role in lipid metabolism [18]. Higher levels of serum remnant-like particle cholesterol (RLP-C), which is the atherogenic lipoprotein, have been found in patients with hypothyroidism and subclinical hypothyroidism compared with matched controls, and their RLP-C concentrations were decreased after levothyroxine therapy [19, 20]. There have been reports that levothyroxine therapy increases plasma LPL [19] and HTGL [19, 20] activities. However, the correlations between serum free thyroid hormone concentrations and LPL, HTGL, and GPIHBP1 concentrations have not been fully elucidated.

Extensive studies on the tissue-specific activities of lipases in animal models and circulating activities and concentrations of lipases in plasma have been conducted to clarify the biological functions of LPL and HTGL [6, 7]. Studies were conducted to specially investigate LPL’s association with exercise because of expression in skeletal muscle. Previous reports have proven that regular exercise lowers serum TG concentrations through increases in muscle and serum LPL activities [7, 21]. In contrast, it has been shown that decreased serum LPL concentrations are associated with metabolic syndrome, diabetes, and coronary atherosclerosis [22–26]. In the case of HTGL, although its pathophysiologic role has been investigated mainly in terms of association with atherosclerosis, by measurement of circulating HTGL activities, studies have indicated contradictory results regarding associations with atherosclerosis [27–31]. In the past, owing to the low sensitivities of assays, previous methods used for measuring circulating activities and concentrations of lipases required heparin administration. Therefore, assay methods based on the monoclonal antibodies against these lipases have been developed recently to achieve sufficient sensitivities for measurement of serum LPL and HTGL concentrations without the need for heparin administration [32–34]. As a result, preheparin serum LPL concentrations can now be measured using a latex particle-enhanced turbidimetric immunoassay with an automated analyzer [35], and preheparin serum HTGL concentrations can be determined using high-sensitivity HTGL enzyme-linked immunosorbent assay (ELISA) [34]. Furthermore, ELISA methods have been developed recently for the measurement of serum concentrations of GPIHBP1 [36].

These methods were used in two recent studies to demonstrate the clinical relevance of preheparin serum concentrations of LPL and HTGL and serum concentrations of GPIHBP1. The first study reported that in patients with coronary artery disease, increases in atherogenic lipoproteins, such as RLP-C, were associated with an increase in preheparin serum HTGL concentration and a decrease in preheparin serum LPL concentration [37]. The second study demonstrated that in obese middle-aged women, a diet and exercise intervention contributed to increased serum GPIHBP1 concentration, but not LPL concentrations, in association with a decrease in body weight and body fat [38]. However, these two studies found that multiple factors influenced lipid metabolism because most participants were older people who had arteriosclerosis or metabolic disorders and took medication, including statins. Furthermore, the relationship between skeletal muscle mass and lipid metabolism, including LPL, GPIHBP1, and HTGL, remains to be elucidated. One effective way to clarify these relationships is to conduct an investigation on young individuals with abundant skeletal muscle who do not have age-related factors. In addition, presentation of these relationships may lead to a clarification of the mechanisms involved in metabolic dysfunctions caused by sarcopenia.

In this study, the aims were to analyze the characteristics of body composition and serum lipid metabolism and investigate the association between skeletal muscle mass and serum concentrations of LPL, GPIHBP1, and HTGL in young Japanese men, which included skeletal muscle-rich wrestling athletes, who did not have metabolic disorders or arteriosclerosis.

## Materials and methods

### Participants

Participants in this cross-sectional study included 111 young, healthy Japanese men who were not taking medications for metabolic diseases. Seventy were amateur wrestlers (age range, 16–26 years) and 41 were college students who did not engage in habitual hard exercise (age range, 18–24 years). In general, wrestling athletes begin to decrease their food intake for weight loss and to exercise hard at approximately 2 weeks before a wrestling tournament with weight categories [39]. In this study, wrestling athletes’ body compositions, serum lipoproteins, lipases, and lipase-associated protein were determined during periods wherein they were not under dietary restrictions or were not undergoing hard practices for a tournament.

Participants provided written informed consent before being included in the study. The ethics committee of Gunma University Graduate School of Medicine approved this study (approval no. 13–36).

### Physical examinations

Physical examinations and blood sample collections were performed in the morning after a 12-h fast. A bioimpedance instrument (InBody 430; InBody Japan, Tokyo, Japan) was used to measure body weight, fat mass, and skeletal muscle mass, with the participant in the standing position. Body mass index (BMI), fat mass index (FMI), and skeletal muscle index (SMI) were calculated respectively as follows: weight/height squared (kg/m^2^), fat mass/height squared (kg/m^2^), and skeletal muscle mass/height squared (kg/m^2^).

### Measurement of thyroid function and serum concentrations of lipoprotein, lipases, and lipase-associated protein

With the participants sitting down, blood samples were collected after an antecubital vein is punctured using 23-G needles. Serum samples were separated by centrifugation (1500×*g*) at 4 °C for 10 min and were stored at − 80 °C until analysis. A chemiluminescent microparticle immunoassay on an Abbott ARCHITECT i2000SR Immunoassay Analyzer (Abbott Laboratories, Abbott Park, Illinois) was used to analyze the serum concentrations of free 3,5,3′-triiodothyronine (FT_3_), free thyroxine (FT_4_), and thyrotropin (TSH). Enzymatic methods using a LABOSPECT 008 automatic analyzer (Hitachi, Tokyo, Japan) were carried out to measure serum total cholesterol (TC), HDL cholesterol (HDL-C), and TG concentrations. The Friedewald formula was used to determine LDL cholesterol (LDL-C) concentrations: LDL-C = TC − HDL-C − TG/5 [40].

LPL latex agglutination turbidimetry (Immuno-Biological Laboratories [IBL], Gunma, Japan) was used to measure serum LPL concentrations [35]. A Human GPIHBP1 Assay Kit (IBL) based on sandwich ELISA was used to determine serum GPIHBP1 concentrations [36]. Finally, the Human Serum HTGL ELISA Kit (IBL) was used to measure serum HTGL concentrations [34].

### Statistical analysis

Because almost all variables were not normally distributed, data are expressed as median values with a 25th–75th percentile range, rather than as mean values with standard deviations. Unpaired Student’s *t* tests or Mann–Whitney *U* tests were used, as appropriate, to identify statistically significant differences between the two study groups. Kruskal–Wallis tests with Bonferroni multiple comparison tests were performed to compare the two groups classified by quartile. Spearman’s correlation analyses were carried out to determine the relationships between serum concentrations of LPL, GPIHBP1, or HTGL and the clinical parameters. Differences and correlations were considered significant when *p* < 0.05. SPSS Statistics version 25.0 (SPSS, Inc., Chicago, Illinois) was used to perform all statistical analyses.

## Results

### Clinical characteristics in control and wrestling athlete participants

The 111 young Japanese men were divided into two groups: control participants (*n* = 41), who did not engage in habitual hard exercise, and wrestling athletes (*n* = 70). Table 1 shows the results of the physical examinations and lipid metabolism analyses in the two groups. Compared with participants in the control group, participants in the athlete group had significantly higher weights (*p* = 0.012), BMI (*p* < 0.001), and SMI (*p* < 0.001) and lower FMI (*p* = 0.024). Similarly, lipid metabolism analyses showed that the athlete group participants had significantly higher serum concentrations of HDL-C (*p* = 0.016), LPL (*p* < 0.001), and GPIHBP1 (*p* < 0.001). Serum concentrations of TG tended to be lower in athletes, although the differences between the two groups were not statistically significant (*p* = 0.288). There were no differences in serum HTGL concentrations and thyroid function tests between the groups. Results of the thyroid function tests showed values that were within reference range in all participants.

**Table 1:** Clinical Characteristics in Control and Wrestling Athlete Participants

	All Participants	Control Participants	Athletes	*p*
	(*n* = 111)	(*n* = 41)	(*n* = 70)	
Age (years)	20 (19.0–21.0)	19 (18.0–20.0)	20 (19.0–21.0)	0.001
Weight (kg)	66.6 (62.3–74.2)	64.2 (58.1–68.4)	68.4 (63.6–75.9)	0.012
BMI (kg/m^2^)	23.7 (22.2–25.6)	21.7 (20.1–24.0)	24.2 (23.2–26.0)	< 0.001
FMI (kg/m^2^)	2.7 (2.3–3.7)	3.2 (2.3–4.9)	2.6 (2.2–3.5)	0.024
SMI (kg/m^2^)	11.9 (11.0–12.5)	10.5 (9.9–11.5)	12.4 (11.9–13.0)	< 0.001
TC (mg/dL)	168 (150–186)	171 (156–186)	167 (145–186)	0.452
LDL-C (mg/dL)	94 (83–110)	100 (85–115)	93 (82–105)	0.282
HDL-C (mg/dL)	56 (51–66)	53 (48–63)	59 (53–67)	0.016
TG (mg/dL)	64 (49–81)	66 (49–101)	64 (49–76)	0.288
LPL (ng/mL)	62.5 (52.5–72.6)	52.3 (47.6–59.0)	67.3 (60.2–76.0)	< 0.001
GPIHBP1 (pg/mL)	986 (853–1150)	934 (742–1039)	1038 (890–1283)	< 0.001
HTGL (ng/mL)	44.1 (33.1–61.1)	43.9 (34.6–61.3)	45.5 (30.0–61.0)	0.525
FT_3_ (pg/mL)	3.22 (3.09–3.39)	3.18 (3.04–3.34)	3.23 (3.10–3.41)	0.115
FT_4_ (ng/dL)	1.04 (0.98–1.10)	1.02 (0.98–1.10)	1.05 (0.97–1.11)	0.318
TSH (μIU/mL)	1.78 (1.24–2.25)	1.84 (1.19–2.12)	1.67 (1.36–2.30)	0.647

Data are expressed as median (25th–75th percentile)

Wrestling athletes were compared with control participants using the unpaired Student’s *t* test or Mann-Whitney *U* test as appropriate

*BMI* body mass index, *FMI* fat mass index, *SMI* skeletal muscle index, *TC* total cholesterol, *LDL-C* low-density lipoprotein cholesterol, *HDL-C* high-density lipoprotein cholesterol, *TG* triglyceride, *LPL* lipoprotein lipase, *GPIHBP1* glycosylphosphatidylinositol anchored high-density lipoprotein binding protein 1, *HTGL* hepatic triglyceride lipase, *FT*_*3*_ free 3,5,3′-triiodothyronine, *FT*_*4*_ free thyroxine, *TSH* thyrotropin

### Association between SMI and serum concentrations of LPL, GPIHBP1, and HTGL

**Fig. 1:**
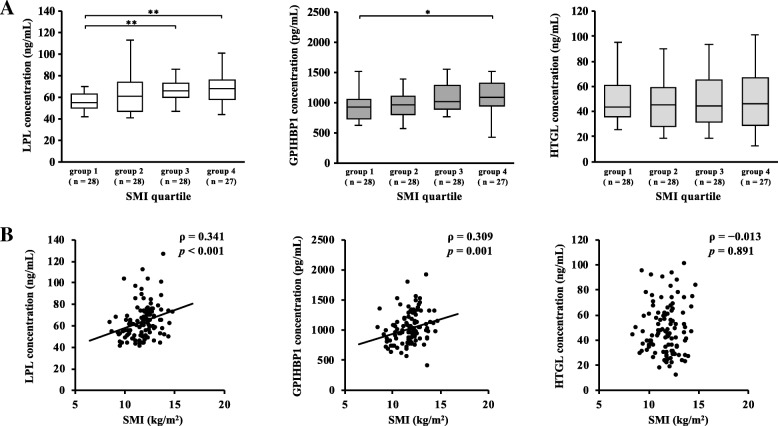
Association between SMI and serum concentrations of LPL, GPIHBP1, and HTGL (*n* = 111 men). **a** Serum LPL, GPIHBP1, and HTGL concentrations between the two groups, classified by SMI quartile (group 1, SMI ≤ 11.0; group 2, 11.0 < SMI ≤ 11.9; group 3, 11.9 < SMI ≤ 12.5; group 4, 12.5 < SMI). Kruskal–Wallis tests with Bonferroni multiple comparison tests were used to compare the two groups (*p < 0.05, ** *p* < 0.01). **b** Results of the Spearman’s correlation analyses between SMI and serum concentrations of LPL, GPIHBP1, and HTGL

Figure 1 shows the associations between SMI and serum concentrations of LPL, GPIHBP1, and HTGL in all participants. Figure 1a shows the comparison of serum concentrations of LPL, GPIHBP1, and HTGL between the SMI groups classified by quartile. The SMI values of each group are as follows: group 1, SMI ≤ 11.0; group 2, 11.0 < SMI ≤ 11.9; group 3, 11.9 < SMI ≤ 12.5; and group 4, 12.5 < SMI. Kruskal–Wallis tests indicate that there were significant differences among the SMI groups in serum concentrations of LPL (*p* < 0.001) and GPIHBP1 (*p* = 0.011). Moreover, Bonferroni multiple comparison tests show that the serum concentrations of LPL in groups 3 and 4 were significantly higher than that in group 1, and serum concentrations of GPIHBP1 in group 4 were significantly higher than that in group 1. There was no significant difference in serum HTGL concentrations among the SMI groups. In addition, results of the Spearman’s correlation analyses show that SMI was positively correlated with LPL (ρ = 0.341, *p* < 0.001) and GPIHBP1 (ρ = 0.309, *p* = 0.001) concentration but was not correlated with HTGL concentration (ρ = − 0.013, *p* = 0.891) (Fig. 1b). Weight-adjusted skeletal muscle mass, calculated as skeletal muscle mass/weight, was also correlated with LPL (ρ = 0.364, *p* < 0.001) and GPIHBP1 (ρ = 0.206, *p* = 0.022) concentration (data not shown).

### Correlation between LPL and GPIHBP1 concentrations and clinical variables

**Fig. 2:**
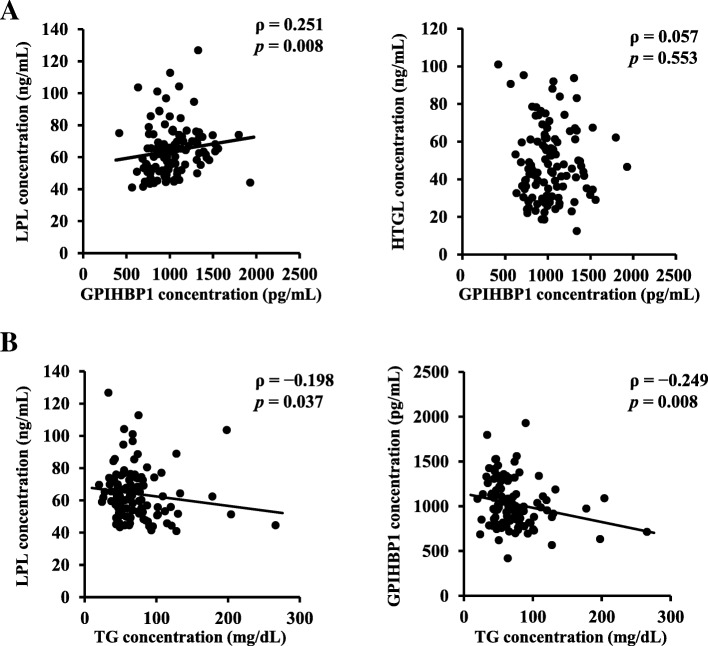
Correlations between serum LPL and GPIHBP1 concentrations and clinical variables (*n* = 111 men). Results of the Spearman’s correlation analyses (**a**) between serum GPIHBP1 concentrations and serum LPL and HTGL concentrations and (**b**) between serum TG concentration and serum LPL and GPIHBP1 concentrations

Figure 2a shows the results of the Spearman’s correlation analyses between GPIHBP1 and LPL or HTGL concentrations in all participants. Results indicate that LPL concentration was positively correlated with GPIHBP1 concentration (ρ = 0.251, *p* = 0.008). In contrast, HTGL concentration was not correlated with GPIHBP1 concentrations (ρ = 0.057, *p* = 0.553). Table 2 shows the correlation between serum concentrations of LPL and GPIHBP1 and other clinical variables in all participants. LPL concentration were positively correlated with SMI and HDL-C (*ρ* = 0.389, *p* < 0.001) and inversely correlated with FMI (ρ = − 0.222, *p* = 0.019) and TG (ρ = − 0.198, *p* = 0.037; Fig. 2b). Moreover, GPIHBP1 concentrations were positively correlated with SMI and inversely correlated with TG (ρ = − 0.249, *p* = 0.008; Fig. 2b). LPL and GPIHBP1 concentrations were not correlated with results of the thyroid function tests.

**Table 2:** Spearman’s correlation analyses between LPL, GPIHBP1, and HTGL concentrations and clinical variables in 111 participants

	LPL		GPIHBP1		HTGL	
Variable (*n* = 111)	ρ	*p*	ρ	*p*	ρ	ρ
Age (years)	0.053	0.577	−0.059	0.542	0.065	0.501
Weight (kg)	0.075	0.432	0.153	0.109	0.065	0.496
BMI (kg/m^2^)	0.123	0.199	0.167	0.081	0.087	0.361
FMI (kg/m^2^)	−0.222	0.019	−0.143	0.135	0.112	0.210
SMI (kg/m^2^)	0.341	< 0.001	0.309	0.001	−0.013	0.891
TC (mg/dL)	−0.003	0.976	−0.167	0.080	0.308	0.001
LDL-C (mg/dL)	−0.079	0.413	−0.064	0.504	0.336	< 0.001
HDL-C (mg/dL)	0.389	< 0.001	0.003	0.974	−0.092	0.337
TG (mg/dL)	−0.198	0.037	−0.249	0.008	0.057	0.551
FT_3_ (pg/mL)	0.161	0.092	0.017	0.862	0.260	0.006
FT_4_ (ng/dL)	0.139	0.146	0.086	0.371	0.035	0.712
TSH (μIU/mL)	0.121	0.204	0.003	0.971	−0.041	0.669

### Correlation between HTGL concentrations and clinical variables

**Fig. 3:**
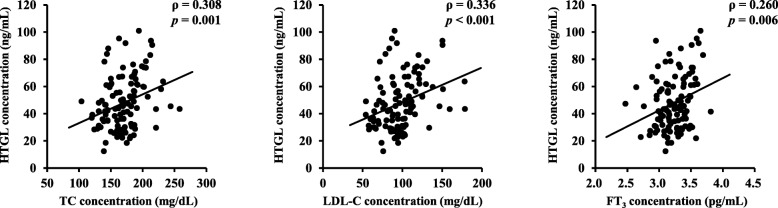
Correlations between serum HTGL concentrations and clinical variables (*n* = 111 men). Results of the Spearman’s correlation analyses between serum HTGL concentration and TC, LDL-C, and FT_3_ concentration

Table 2 and Fig. 3 show the results of the Spearman’s correlation analyses between HTGL concentrations and other clinical variables in all participants. HTGL concentration was positively correlated with TC (ρ = 0.308, *p* = 0.001), LPL-C (ρ = 0.336, *p* < 0.001), and FT_3_ (ρ = 0.260, *p* = 0.006) concentrations but was not correlated with FT_4_ and TSH concentrations. FT_3_, FT_4_, and TSH concentrations were not correlated with any data from the physical examination or lipoprotein analyses (data not shown).

## Discussion

The analyses in this study show the associations between skeletal muscle mass and lipid metabolism, including LPL, GPIHBP1, and HTGL, in young Japanese men. Significantly high serum concentrations of preheparin LPL and GPIHBP1 were found in wrestling athletes with abundant skeletal muscle mass. Participants with higher SMI showed significantly higher serum concentrations of LPL and GPIHBP1. Moreover, in all participants, serum concentrations of LPL and GPIHBP1 correlated positively with SMI and inversely with TG concentrations. Serum HTGL concentration was positively correlated with TC, LDL-C, and FT_3_ concentrations but not was correlated with skeletal muscle mass.

Previous studies have shown that preheparin serum LPL concentrations were positively correlated with HDL-C concentration and inversely correlated with TG concentration [22, 37, 41–43]. In addition, it has been reported that LPL concentration is inversely correlated with intra-abdominal visceral fat accumulation, as evaluated by computed tomography [22], and with the number of metabolic syndrome symptoms [23]. In agreement with these studies, we observed correlations of LPL with lipoprotein concentrations and FMI. Some studies have identified several GPIHBP1 gene missense mutations that cause chylomicronemia [8, 10–15]. These mutations block the ability of GPIHBP1 to bind LPL and shuttle it to the capillary lumen owing to the disruption to fold the lymphocyte antigen 6/urokinase-type plasminogen activator receptor domain that binds LPL with high affinity [10–15]. In addition, recent reports have identified the presence of GPIHBP1 antibodies that inhibit the ability of GPIHBP1 to bind and transport LPL in patients with hypertriglyceridemia [16]. The positive correlation between GPIHBP1 and TG concentrations in this study agrees with the relevance reported in these previous studies. In addition, in agreement with a previous study [37], there was a positive correlation between GPIHBP1 concentration and LPL concentration, but there was no correlation between GPIHBP1 concentration and HTGL concentration. On one hand, several studies on humans have revealed that physical exercise leads to an increase in LPL activity and mRNA in the exercising muscle [44–48] and to an increase in postheparin plasma LPL activity and preheparin serum LPL concentration [49, 50]. On the other hand, one study [38] reported an association between GPIHBP1 concentration and physical exercise, wherein reduction in weight as a result of a diet and exercise intervention increased serum GPIHBP1 concentration, but not serum LPL concentration, in Japanese women who were obese. However, the relationship between LPL and GPIHBP1 and skeletal muscle mass, including sarcopenia, has not been fully understood. In this study, there were significantly higher LPL and GPIHBP1 concentrations in wrestling athletes than in control participants. This difference may be affected not only by the quantity but also the quality of athletes’ muscle with continuing habitual trainings, but the multiple comparison analysis and the correlation analysis among all participants, including controls, also indicated that the participants with higher SMI had higher LPL and GPIHBP1 concentrations as well. To our knowledge, this is the first study to report on the relevance of the relationship between skeletal muscle mass and serum concentration of GPIHBP1, as well as serum concentrations of LPL. The results of this study suggest that increased skeletal muscle mass results in effective energy use through the promotion of TG-rich lipoprotein hydrolysis through elevation of serum LPL and GPIHBP1 concentrations. Conversely, unlike LPL, GPIHBP1 concentration was not correlated with FMI and HDL-C concentrations. Future studies are needed to explore these distinctions in correlations through clinical and molecular analyses and to investigate the association between these proteins and the qualities of skeletal muscle, such as slow-stich muscle and fast-stich muscle.

The inverse relation between HTGL activities and the buoyancies and sizes of LDL and HDL particles has been shown in one study [9]. In addition, in the last decade, a number of studies have reported on increases and decreases in HTGL activities associated with atherosclerosis [27–31]. In a recent study [37] using high-sensitivity ELISA for patients with coronary artery diseases, preheparin serum HTGL concentrations were positively correlated with small dense LDL-C (sdLDL-C) and RLP-C concentrations, but not with LDL-C or large-buoyant LDL-C concentrations. However, because most participants in that study were taking statins, only the positive correlations with sdLDL-C, which is unlikely to be affected by statins, were considered. In contrast, in the young participants in this study, age, medication use, or anamnestic factors had no effect on lipid metabolism, which were likely reflected by the positive correlation between HTGL and LDL-C concentrations. Moreover, in this study, there were no differences in serum HTGL concentrations between the athletes and the control participants, and there was no correlation between the HTGL concentration and skeletal muscle mass. This suggests that HTGL is produced by hepatocytes, but not by skeletal muscle cells [9, 17].

Previous studies have demonstrated that in patients with hypothyroidism who were treated with levothyroxine, increased HTGL activities were associated with decreases in serum RLP-C concentration [19, 20]. Moreover, one study [51] showed that T_3_ increased the posttranslational HTGL expression in HepG2 cells. However, no direct correlations between HTGL activities and thyroid hormone concentrations were demonstrated. In this study, there was a significant, positive correlation between FT_3_ and preheparin serum HTGL concentrations in young men with euthyroid state. This correlation provides support for the relationships between HTGL activities and thyroid hormones shown in previous molecular and clinical studies. In this study, however, there were no correlations between serum HTGL concentrations and FT_4_ and TSH concentrations. The differences in the correlations with HTGL concentration may be attributable to more potent physiologic activity of T_3_ than that of T_4_. Our results suggest that HTGL seems to accelerate the synthesis of LDL-C in proportion to serum FT_3_ concentration. Future studies are needed to determine the detailed effect of thyroid hormone on lipid metabolism, including HTGL, in various thyroid states.

Limitations of this study should be acknowledged, which include the cross-sectional design of the study and the relatively small sample size. One reason for the small sample size is that only men were enrolled in this study. Women were not included because they have menstrual cycles and different body compositions that may have an effect on lipid metabolism. Although there were no participants with sarcopenia in this study who had skeletal muscle mass less than 20 kg and SMI less than 7.0 kg/m^2^, which are cutoff points provided by the European Working Group on Sarcopenia in Older People 2 [52], we focused enrollment on many young wrestling athletes with a large amount of skeletal muscle in the same condition without extreme dietary restrictions and hard practices. Moreover, the control participants were significantly younger than the wrestling athletes, but this may not have been a serious confounder as there was an almost unremarkable difference between the ages of the two groups, and no participant had age-related factors, such as medication use or metabolic diseases. These assertions can also be supported by analyses conducted for other lipid markers, such as RLP-C and sdLDL-C. Moreover, further studies are needed to confirm our hypothesis by evaluating more markers of total energy metabolism in a larger cohort that includes women, elderly people, individuals with sarcopenia, and the like.

## Conclusions

In conclusion, the serum concentrations of LPL and GPIHBP1 were significantly high in skeletal muscle-rich participants and were positively correlated with skeletal muscle mass in young Japanese men. These results suggest that increases in skeletal muscle mass lead to improvements in energy use by promoting TG-rich lipoprotein hydrolysis through increases in circulating LPL and GPIHBP1 concentrations. In contrast, high serum concentration of HTGL seems to increase the synthesis of serum LDL-C in proportion to FT_3_ concentration, regardless of skeletal muscle mass.

## Abbreviations

BMI: Body mass index
ELISA: Enzyme-linked immunosorbent assay
FMI: Fat mass index
FT_3_: Free 3,5,3′-triiodothyronine
FT_4_: Free thyroxine
GPIHBP1: Glycosylphosphatidylinositol anchored high-density lipoprotein binding protein 1
HDL-C: High-density lipoprotein cholesterol
HTGL: Hepatic triglyceride lipase
LDL-C: Low-density lipoprotein-cholesterol
LPL: Lipoprotein lipase
RLP-C: Remnant-like particles-cholesterol
sdLDL-C: Small dense low-density lipoprotein-cholesterol
SMI: Skeletal muscle index
TC: Total cholesterol
TG: Triglyceride
TSH: Thyrotropin
